# Diesel exhaust exposure alters the expression of networks implicated in neurodegeneration in zebrafish brains

**DOI:** 10.1007/s10565-021-09618-9

**Published:** 2021-05-31

**Authors:** M. Saeid Jami, Hiromi Murata, Lisa M. Barnhill, Sharon Li, Jeff M. Bronstein

**Affiliations:** 1 Department of Neurology, David Geffen School of Medicine At UCLA, 710 Westwood Plaza, Los Angeles, CA 90095 USA; 2 Molecular Toxicology IDP, David Geffen School of Medicine At UCLA, Los Angeles, CA USA

**Keywords:** Air pollution, Dementia, Parkinson’s disease, Alzheimer’s disease, Transcriptomics, Proteomics

## Abstract

Neurodegenerative diseases are a major cause of disability in the world, but their etiologies largely remain elusive. Genetic factors can only account for a minority of risk for most of these disorders, suggesting environmental factors play a significant role in the development of these diseases. Prolonged exposure to air pollution has recently been identified to increase the risk of Alzheimer’s and Parkinson’s diseases, but the molecular mechanisms by which it acts are not well understood. Zebrafish embryos exposed to diesel exhaust particle extract (DEPe) lead to dysfunctional autophagy and neuronal loss. Here, we exposed zebrafish embryos to DEPe and performed high throughput proteomic and transcriptomic expression analyses from their brains to identify pathogenic pathways induced by air pollution. DEPe treatment altered several biological processes and signaling pathways relevant to neurodegenerative processes, including xenobiotic metabolism, phagosome maturation, and amyloid processing. The biggest induction of gene expression in brains was in Cyp1A (over 30-fold). The relevance of this expression change was confirmed by blocking induction using CRISPR/Cas9, which resulted in a dramatic increase in sensitivity to DEPe toxicity, confirming that Cyp1A induction was a compensatory protective mechanism. These studies identified disrupted molecular pathways that may contribute to the pathogenesis of neurodegenerative disorders. Ultimately, determining the molecular basis of how air pollution increases the risk of neurodegeneration will help in the development of disease-modifying therapies.

**Graphical abstract:**

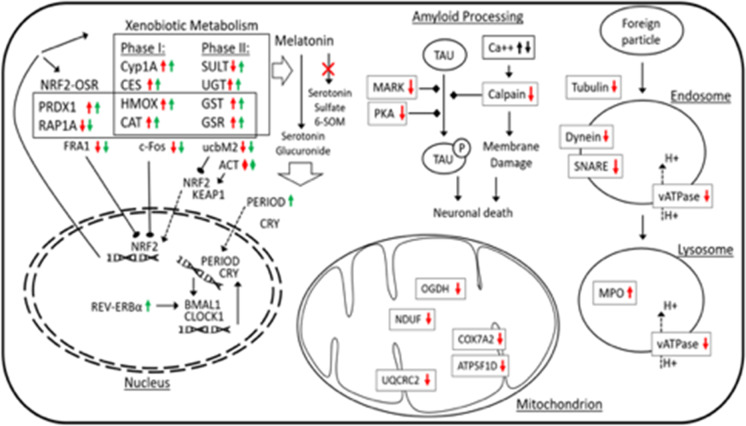

**Supplementary Information:**

The online version contains supplementary material available at 10.1007/s10565-021-09618-9.

## Introduction

Air pollution is a major contributor to mortality and is associated with respiratory disease, heart disease, stroke, lung cancer, and diabetes. Emerging epidemiological evidence supports a link between air pollution exposure and the development of neurodegenerative diseases, including Alzheimer’s and Parkinson’s diseases (AD and PD) (Fu et al. 2019). There is a paucity of research investigating the mechanisms by which air pollution may increase the risk of AD and PD, but the few that do exist are supportive of a causal relationship (Fu et al. 2019).

The accumulation of protein inclusions in the brain is a universal feature of neurodegenerative disorders and is likely a common pathway leading to neuronal dysfunction and death. For example, the formation of amyloid-beta (A-beta) and tau structures are the pathological hallmarks of AD and α-synuclein (α-syn) aggregates or Lewy Bodies in PD (Ross and Poirier 2004; Woulfe 2008). Alterations in proteostasis appear to at least partially underlie the formation of these aggregates, but the precise mechanisms are still unknown and may vary in individuals depending on genetic and environmental risk factors. We do know that increased expression or decreased degradation of A-beta or α-syn can lead to AD and PD (Bostancıklıoğlu 2019; Johnson et al. 2019). Other common features in neurodegenerative disorders are inflammation and oxidative stress. Activated microglia, the inflammatory cells in the CNS, can be readily appreciated in autopsies of AD and PD brains and likely contribute to the pathogenesis of neurodegeneration (Kannarkat et al. 2013). This inflammation and possibly mitochondrial dysfunction are thought to lead to oxidative stress which is also appreciated in autopsied brains (Picca et al. 2020).

The majority of mechanistic studies on air pollution and neurodegeneration to date have focused on inflammation (Jayaraj et al. 2017). Air pollution appears to increase both CNS inflammation and oxidative stress in animal and human brains (Calderón-Garcidueñas et al. 2004, 2007, 2008a, b and c; Levesque et al. 2011a, b, 2013; Moulton and Yang 2012; Mumaw et al. 2017; Yokota et al. 2013a, b). Of particular interest are the findings that air pollution increases the expression of some inflammatory genes in the olfactory bulb (OB) of mice, a brain region where PD pathology is seen very early in the disease (Levesque et al. 2011a, b; Yokota et al. 2013a). Inflammation was also seen in dogs living in urban areas compared to those living in rural areas, and the authors speculated that these changes were due to high levels of air pollution (Calderón-Garcidueñas et al. 2003). The same authors also suggested that α-syn accumulated in the brains of people living in cities due to air pollution (Calderón-Garcidueñas et al. 2004, 2008a, b). In a recent study in a zebrafish model, exposure to DEPe was reported to cause neurotoxicity and a significant decrease in neuron number by disrupting autophagy (Barnhill et al. 2020). DEPe contains many of the toxic components of air pollution and is commonly used to model exposure in cell culture systems (Costa et al. 2014; Hesterberg et al. 2010; Levesque et al. 2011b). Autophagy is an essential intracellular mechanism for the removal and eradication of misfolded proteins and damaged organelles. Indeed, precise autophagic activity is necessary for cellular homeostasis, and dysfunctional autophagy in neurons leads to altered survival and neurodegeneration (Kesidou et al. 2013).

There are clear limitations in many of these studies, but they all suggest that air pollution exposure increases the risk of AD and PD. Our understanding of the mechanisms by which pollution increases this risk is limited, but it is clear that ultrafine particles and several components of air pollution can enter the brain either directly through the olfactory bulb or through the lungs via the bloodstream (Kilian and Kitazawa 2018). These particles are composed of a carbon core and adsorbed compounds such as polycyclic aromatic hydrocarbons, metals, nitrate, sulfate, and other elements and constitute a significant proportion of air pollutants, especially in urban areas. Many of these compounds have been implicated in oxidative stress and cellular toxicity adding plausibility to a causal association of air pollution and neurodegeneration.

Detailed multidisciplinary investigations, including cellular and animal models, can give insight into the causes of disease and direct future therapeutic approaches (Cannon and Greenamyre 2011). Among the model organisms used for in vivo studies, *Danio rerio* (zebrafish) is a powerful model to study toxicology, molecular genetics, toxicogenomics, and drug discovery. Most biological pathways are highly conserved in vertebrates, and the majority of functional human genes have homologs in zebrafish (Vaz et al. 2018). In addition, zebrafish embryos develop rapidly and independently, allowing for analysis at any desired developmental stage. These features, together with rapid reproduction and low cost of husbandry, make zebrafish a favorable model for studying the toxic effects of environmental exposures.

In order to better understand the molecular mechanisms behind the pathogenesis of the disease, a wide range of high throughput gene expression profiling approaches has been developed. Mass spectrometry–based proteomics and high-throughput RNA sequencing hold particular promise to identify biological pathways of interest. These techniques have been widely used to understand the response of cellular and animal models to environmental stimuli (Duan et al. 2017; García-Estrada et al. 2013; Jami et al. 2014a, b, 2015; Kosalková et al. 2012). In this study, we have pursued expression analyses of the effects of DEPe treatment on both the proteome and transcriptome profiles within the heads of zebrafish embryos. Isolation of samples from the head that is mainly composed of the brain tissue allowed for obtaining more tissue-specific profiles and minimized effects originating from other tissues. We describe here altered gene expression and protein profiles in a number of pathways implicated in neurodegeneration in the heads of DEPe-exposed zebrafish. These results provide new insights into the possible mechanisms by which air pollution increases the risk of AD and PD.

## Material and methods

### Fish treatment and CRISPR/Cas9-mediated gene knockdown

All studies were approved by the UCLA Animal Rights Committee. Zebrafish (AB) were bred by light stimulation for 1 h, and a total number of 200 eggs were incubated for 24 h at 28 °C. The resulting embryos were dechorionated in pronase (2 mg/ml) and treated with either DEPe (Standard Reference Materials, NIST, Gaithersburg, MD) at a final concentration of 20 μg/ml or vehicle until 5 days post-fertilization (DPF). This concentration was selected since it resulted in neuron loss as previously reported but without significant mortality (Barnhill et al. 2020).

Cyp1A knockdown fish were prepared as follows. Using tools available at http://crispr.mit.edu, the CRISPR RNA (Cyp1A-crRNA) was designed based on the location of suitable PAM sites in the first exon of Cyp1A gene. The best design was purchased from IDT DNA (www.idtdna.com) together with the standard CRISPR-Cas9 tracrRNA. The crRNA for Cyp1A gene and tracrRNA were separately resuspended in 10 mM Tris–EDTA to yield a 100 μM final concentration. Then, tracrRNA was combined with crRNA in 1 × duplex buffer (100 mM potassium acetate; 30 mM HEPES, pH 7.5; IDT DNA) to yield a final concentration of 10 μM. The mixture was heated to 95 °C for 5 min on a boiling water bath and slowly cooled to room temperature to allow for the annealing of the complementary nucleotide sequences in crRNA and tracrRNA and the formation of functional guide RNA (gRNA). A volume of 3 μL of annealed gRNA was then combined with the same volume of a donor single-strand DNA (ssDNA; 200 μM) as the template sequence for mutant Cyp1A. An amount of 3 μg of Cas9 Nuclease (IDT) in 1 × injection buffer (5 mM KCl; 0.1 M sodium phosphate, pH 6.8) was added to the mixture to form the final CRISPR/Cas9 ribonucleoprotein (Cyp1A-RNP) complexes. For the preparation of the Scramble RNP (SC-RNP), the specific Cyp1A-crRNA was substituted by a commercial Scramble-crRNA with no target on the genome. The sequence of all oligonucleotides used in this work is listed in Supplementary Table 1.

Eggs were injected under a stereomicroscope with around 3 nL of RNP injection mixture. Five biological replicate injections (each replicate included 400–600 eggs injected with Cyp1A-RNP and 100–200 injected with SC-RNP) were performed, and all microinjections were completed within 60 min. Eggs were placed into 100 mm plastic Petri dishes and incubated at 28 °C in egg water. The survival rate of embryos was monitored for up to 7 days. Images of surviving fish were reviewed for scoring malformation. Each larva was scored in a blinded manner for the presence of 4 developmental malformations including head malformation, tail malformation, cardiac edema, and yolk edema. Larvae with all 4 malformations would be scored 4, whereas normal developing larvae would score 0. The statistical T-test was used to evaluate the significant differences.

### Protein sample preparation

In order to prepare protein samples, the heads from 80 to 100 anesthetized larvae (5 dpf) were carefully isolated and washed with PBS before transferring to lysis buffer which consisted of 15% precooled TCA/acetone containing 0.07% betamercaptoethanol (ME) and protease inhibitor cocktail (needs manufacturer) in a total volume of 500 μl. After brief homogenization, proteins were precipitated for 12 h at − 20 °C, followed by centrifugation at 12,000 rpm for 5 min at 4 °C. The supernatant was removed, and the protein pellet was washed twice with the cold acetone (containing 0.07% ME and protease inhibitor cocktails). The final protein pellet was dissolved in lysis buffer containing 7.0 M urea, 2.0 M thiourea by sonication on ice. The solubilized protein samples were centrifuged at 15,000 rpm for 10 min at 4 °C to precipitate insoluble particles, and the concentration of the final protein samples was measured using the Bradford method.

### TMT-based high throughput proteomics analysis

Samples were reduced, alkylated, and digested by the sequential addition of trypsin and lys-C proteases. Peptides were then labeled using 10-plex TMT isobaric tags according to the manufacturer's instructions. Labeled samples were mixed and then fractionated offline using high pH reversed-phase chromatography. Individual fractions were then analyzed by LC–MS/MS using online reversed-phase chromatography and tandem mass spectrometry on a Thermofisher Fusion Lumos mass spectrometer. Data were acquired using the synchronous precursor selection-based MS3 method, as previously described (McAlister et al. 2014). Database searching and the extraction of TMT reporter ion information were performed using the MaxQuant software platform (Cox and Mann 2008). The comparison of TMT data across samples was performed using MSStats (Choi et al. 2014).

### Transcriptomic analysis

Approximately 80–100 heads were isolated from anesthetized embryos (120 h), washed with PBS, and subjected to total RNA extraction using Trizol (Sigma Aldrich, Saint Louis, USA) reagent following the manufacturer’s instructions. The quality and quantity of RNA were evaluated using a NanoDrop 2000 spectrophotometer (Thermo Scientific, MA) and further by Agilent 2100 Bioanalyzer to assure the minimum concentration of 50 ng/μl and RNA integrity number (RIN) of 8. Library preparation was then performed using an Illumina HiSeq4000 according to following the protocol: “TruSeq Stranded Total RNA Library Prep workflow with Ribo-Zero Gold,” and the samples were sequenced at the following conditions: “Paired End run, R1 = 75 cycles (antisense strand), Index = 8 cycles, R2 = 75 cycles (sense strand).”

### Data analysis

Both datasets for proteomic and transcriptomic studies were simultaneously uploaded and analyzed using the online tool Metascape, which allows for gene annotation for various species, including *Danio rerio* (Zhou et al. 2019).

In order to evaluate the contribution of either proteome or transcriptome profile alteration in specific pathways, data were analyzed through the use of Ingenuity Pathways Analysis (IPA) software (Krämer et al. 2014). Data sets containing gene or protein identifiers and corresponding expression values were uploaded into the application. Each identifier was mapped to its corresponding object in Ingenuity’s knowledge base. An expression alteration cutoff of 1.3-fold was set to identify molecules whose expression was differentially regulated. Functional analysis identified the biological functions and/or diseases that were most significant to the data set. Molecules from the dataset that met the cutoff and were associated with biological functions were considered for the analysis. Right-tailed Fisher’s exact test was used to calculate a p-value determining the probability that each biological function and/or disease assigned to that data set is due to chance alone.

### Pathway analysis

To evaluate the contribution of either proteome or transcriptome profile alterations in specific pathways, both datasets were uploaded to the IPA software (Krämer et al. 2014). Top significant altered canonical pathways were then further evaluated and interpreted using PCR and western blotting as described below.

### Western blotting

A total of 25 µg of proteins was loaded on a 12% NuPAGE (Novex, CA) and transferred onto PVDF membranes (Novex, CA) as described (Mahmoudian-Sani et al. 2017; Rafiee et al. 2019). Membranes were blocked with 5% skim milk in Tris-buffered saline and 0.01% tween 20 (TBST buffer) for 30 min at room temperature and then incubated overnight at 4 °C with either rabbit polyclonal anti-Cyp1A1 (Abcam, CA) or mouse monoclonal anti-GAPDH (Abcam, CA), diluted in TBST buffer containing 1% skim milk. After washing in TBST, the blots were incubated with secondary donkey anti-rabbit-HRP (Abcam, CA) or goat anti-mouse-HRP (Santa Cruz, CA) antibodies for 2 h, followed by development with Pierce™ ECL Plus western blotting substrate (Thermo Fisher Scientific, USA). The bands were visualized by imaging using a LI-COR Scanner and analyzed via densitometry (LI_COR Biosciences, NE).

### Real-time PCR

Total RNA was isolated from the heads using TRIzol reagent (Sigma Aldrich, Saint Louis, USA) following the manufacturer’s instructions and then measured using a NanoDrop 2000 spectrophotometer (Thermo Scientific, MA). An amount of 1 μg of each RNA sample was reverse-transcribed using iScript™ reverse transcription supermix (Bio-Rad, CA), and real-time PCR was performed using SsoAdvanced Universal SYBR Green supermix (Bio-Rad, CA), and the primers are listed in Supplementary Table 1. Relative expression levels were calculated using the 2^−ΔΔCT^ method, and the statistical T-test was used to evaluate the significant differences.

## Results and discussion

Proteomic and transcriptomic analyses are two major tools for understanding the molecular mechanisms underlying disease processes and response to environmental stimuli (Duan et al. 2017; García-Estrada et al. 2013; Jami et al. 2014a, b, 2015; Kosalková et al. 2012). Here, we performed deep expression analyses at both the transcriptomic and proteomic levels in the heads of zebrafish embryos exposed to DEPe. The heads, composed of mostly brain tissue, were isolated in order to eliminate the expression profiles of other tissues because we are interested in determining intrinsic pathological pathways of the CNS.

### The expression profile of the heads of zebrafish embryos

**Fig. 1:**
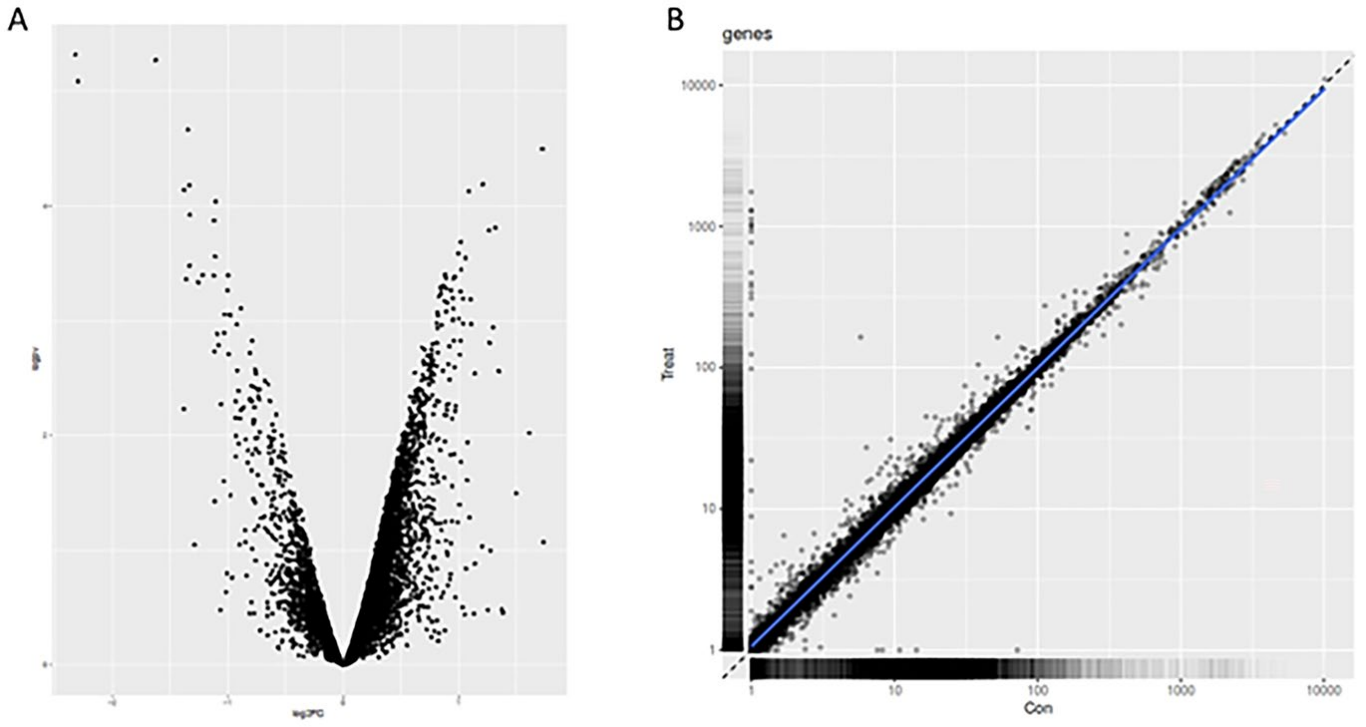
Visualization of expression analyses at proteome **A** and transcriptome **B** levels. A total of 11,172 proteins were identified from the TMT-labeled samples, among which 141 proteins were significantly upregulated and 607 downregulated **A**. The RNA-seq analysis detected 14,748 hits with 367 upregulated and 149 downregulated targets **B**

Profile analysis yielded 11,172 detected proteins and 14,748 mRNA targets out of more than 26,000 coding genes (Howe et al. 2013). Among the 11,172 proteins identified from the TMT-labeled samples (Supplementary Table 2), 141 proteins were significantly upregulated, and 607 downregulated (Supplementary Table 3 and Fig. 1a). Similarly, 367 transcripts were upregulated, and 149 were downregulated among the 14,748 transcripts (Supplementary Table 4) detected in the RNA-seq analysis (Fig. 1b and Supplementary Table 5).

In most cases, the findings from the upregulated proteomic and transcriptomic analyses were consistent. For instance, top highly upregulated proteins include cytochrome P450 Cyp1a, Cyp1c1, Guanine nucleotide-binding protein subunit gamma, Annexin, Plexin B2b short isoform, Dehydrogenase/reductase (SDR family) member 13-like 1, S-antigen of retina/pineal gland (arrestin) b, and Sulfotransferase 6B1. Likewise, genes with the highest transcriptomic upregulation include cytochrome P450 (Cyp1a), chemokine (C–C motif) ligand 27a, aryl-hydrocarbon receptor repressor A, cytochrome P450 (Cyp1b), and Rh family C glycoprotein.

Downregulated proteins and transcripts, on the other hand, were not always highly correlated. For example, Complexin 2, Spectrin alpha, Cardiac myosin light chain-1, SEC23 interacting protein, ATP synthase membrane subunit EA, Cytochrome b, and NAD-dependent protein deacetylase are among the top highly downregulated proteins, while the retinal outer segment membrane protein 1a, solute carrier family 5 (iodide transporter), FBJ murine osteosarcoma viral oncogene homolog B, complexin 4c, FOS-like antigen 1a, opsin 1, and v-fos show the highest downregulation of transcription (Tables 1 and 2).

**Fig. 2:**
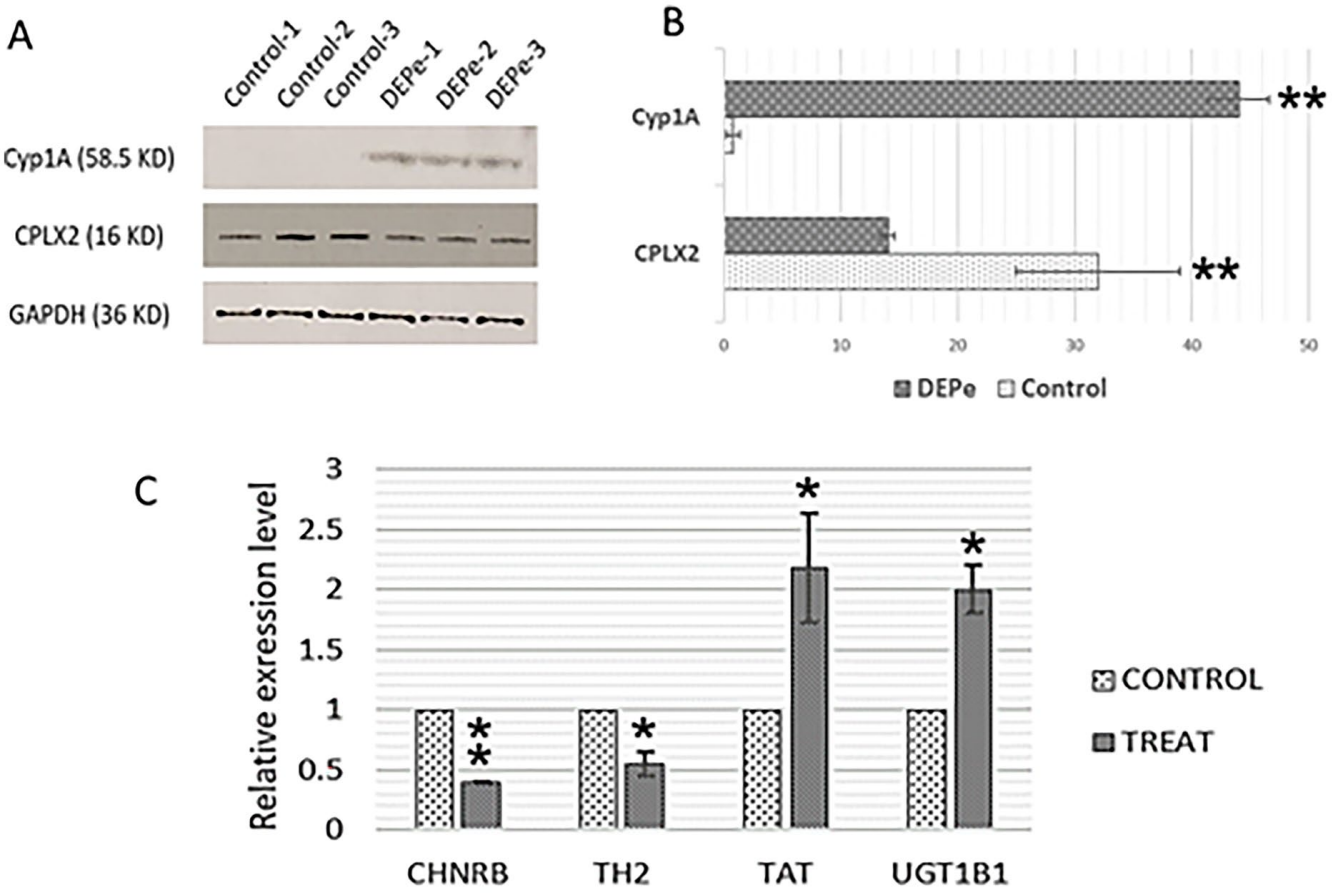
Validation of proteomic and transcriptomic findings. **A** and **B** Upregulation of Cyp1A protein and downregulation of Complexin 2 (CPLX2) was confirmed via western blot considering GAPDH as an internal control. **C** Higher levels of TAT and UGT1B1 transcription and lower levels of CHNRB and TH2 were also confirmed by qPCR using Elf-alpha as the internal control. The asterisks indicate statistical significance (∗ ∗  = p < 0.01, ∗  = p < 0.05)

Antibodies recognizing zebrafish proteins are limited, but we did confirm a sample of these changes using western blot analysis. The upregulation of Cyp1A protein and downregulation of Complexin 2 (CPLX2) were confirmed using GAPDH as a loading control (Fig. 2a). Higher levels of TAT and UGT1B1 transcription and lower levels of CHNRB and TH2 were also confirmed by qPCR using Elf-alpha as the internal control (Fig. 2b).

### Gene annotation

**Fig. 3:**
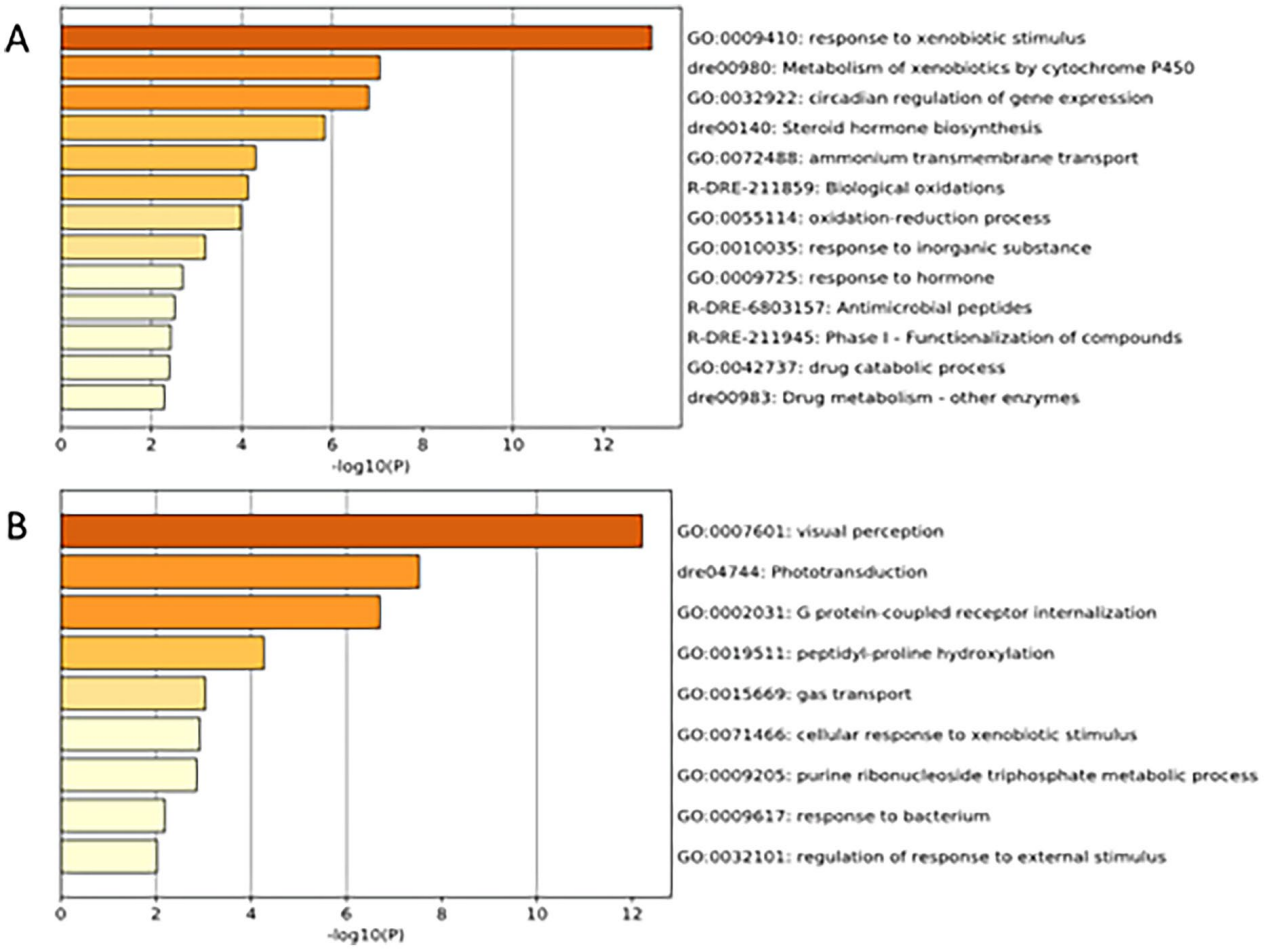
The Metascape online tool allows for gene annotation for *Danio rerio* using combined proteomics and transcriptomics alterations. Several processes such as response to xenobiotic stimulus, metabolism of xenobiotics by cytochrome P450, and circadian regulation of gene expression were found induced upon DEPe treatment **A**, while “Visual perception,” “Phototransduction,” and “G protein-coupled receptor internalization” were suppressed **B**

There are a number of online bioinformatic tools and software that can provide useful information on gene annotation. Among them, Metascape, with the capability of gene annotation for *Danio rerio* (Zhou et al. 2019), was used in this work. Both datasets for proteomic and transcriptomic studies were simultaneously uploaded and analyzed using this online tool. After combining the output of both proteomic and transcriptomic alterations in the software, several processes such as response to xenobiotic stimulus, metabolism of xenobiotics by cytochrome P450, and circadian regulation of gene expression were found induced upon DEPe treatment (Fig. 3a). This analysis also suggested suppressed levels of biological processes such as “Visual perception,” “Phototransduction,” and “G protein-coupled receptor internalization.” The alterations in vision-related expression profiles were not unexpected since DEPe treatment during early development resulted in smaller eyes (data not shown).

### Xenobiotic metabolism signaling

Xenobiotics, which are foreign natural or synthetic chemical compounds, can trigger the cellular stress response, leading to differentiation, proliferation, apoptosis, or necrosis. Indeed, the body needs to actively protect itself against xenobiotics, and also toxic endogenous compounds and their metabolites, via the expression of enzymes and transporters involved in their elimination and detoxification. These enzymes are classified into three groups: Phase I enzymes (CYP, ALDH, FMO) which introduce polarity into the xenobiotics; Phase II enzymes (UGT, GST, SULT) which introduce hydrophilicity via conjugation of hydrophilic molecules such as sulfate, glucuronic acid, and glutathione to the xenobiotics; and Phase III enzymes (MDR1, OATP2, MRP) that transport the xenobiotics or conjugates formed during Phase II to the extracellular area. These enzymes are induced through signaling cascades involving specific receptors (CAR, PXR, AHR) and MAPK-mediated activation of transcription factors (NRF2, MAF) (Omiecinski et al. 2011).

In the absence of activators, the constitutively active receptor (CAR) is located in the cytoplasm as a complex with CCRP and HSP90. But when an activator is present, CAR translocates into the nucleus and binds to RXRα to form a heterodimer and further binds to several variants of the repeat motif, such as DR3, DR4, ER6, and ER8 and contributes to gene expression regulation (Supplementary Table 2).

**Fig. 4:**
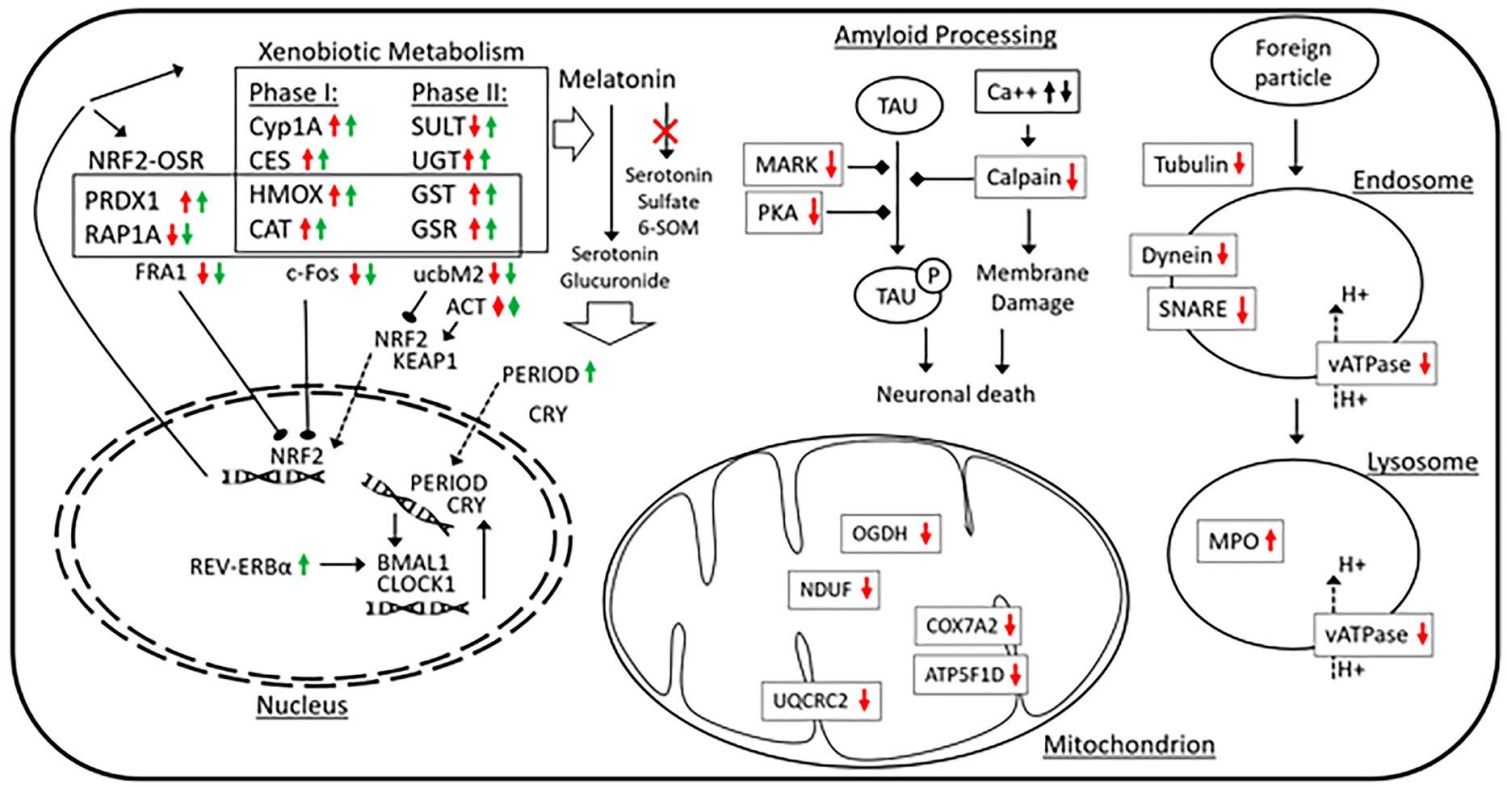
Summarization of biological events during DEPe treatment. Red and green arrows indicate the alterations at protein and transcript levels, respectively

Our proteomics analysis revealed the activation of xenobiotic metabolism. Indeed, the clear upregulation CYP1A1, CYP3A7, HMOX1 (heme oxygenase 1), CAT (catalase), and CES1 (carboxylesterase 1) show the induction of Phase I metabolism, while increased expression of UGT1A1 (UDP glucuronosyltransferase family 1 member A1) and GSTP1 (glutathione S-transferase pi 1) indicates the activation of Phase II metabolism. Interestingly, downregulation of sulfate transferases such as SULT1A1 (sulfotransferase family 1A member 1), SULT1C2 (sulfotransferase family 1C member 2), and SULT2B1 (sulfotransferase family 2B member 1) suggests that increasing hydrophilicity in Phase II metabolism tends to preferably occur via conjugation with glucuronic acid and glutathione, but not sulfate conjugation (Fig. 4).

The results of the transcriptomic study are quite consistent, as CYP1A, CYP1B, CYP3A7, GSTO1, GSTP1, and UGT1A1 are all upregulated at the RNA levels. However, SULT2B1 shows upregulation at the RNA level (while underrepresented at the protein levels). This may be due to the involvement of other mechanisms that induce the degradation or inactivation of sulfate transferase enzymes upon treatment with DEPe. What is particularly surprising is that these changes in xenobiotic metabolism occurred in the heads (presumably brains) of the fish. Expression of xenobiotic genes has been described in the nervous system of mammals, but this is the 1st report of them in zebrafish brains (McMillan and Tyndale 2018).

These findings are consistent with a similar study conducted by Shankar and co-workers who tested the effects of different groups of environmental polycyclic aromatic hydrocarbons (PAHs) on the development of embryos and further evaluated the impact of each treatment regimen on the transcriptome profile. On the whole body of 48 h post-fertilization (hpf) embryos, they showed that the upregulation of Cyp1a at both transcription and protein levels to be an early reliable biomarker of xenobiotic AHR activation and downstream transcriptomic changes (Shankar et al. 2019).

### The NRF2-mediated oxidative stress response

Oxidative stress can trigger apoptosis and necrosis and is believed to be involved in neurodegeneration (Ahmadinejad et al. 2017; Jami et al. 2014b, 2015). A major cellular defense response to oxidative stress is the induction of antioxidant and detoxifying enzymes. Nuclear factor-erythroid 2-related factor 2 (Nrf2) binds to promoter antioxidant response elements (ARE) and activates their transcription. Inactive Nrf2 is associated with an actin-binding protein Keap1 and is retained in the cytoplasm. Triggered by oxidative stress, Nrf2 is phosphorylated in response to the protein kinase C, phosphatidylinositol 3-kinase, and MAP kinase pathways. The phosphorylated Nrf2 can then translocate to the nucleus and bind AREs to transactivate detoxifying and antioxidant enzymes (Supplementary Table 3) (Ma 2013).

The results of proteomics comparison revealed the upregulation (overrepresentation) of ACTG2 (actin, gamma 2), CAT (catalase), GSR (glutathione-disulfide reductase), GST (glutathione S-transferase), HMOX1 (heme oxygenase 1), and PRDX1 (peroxiredoxin 1), as well as the downregulation of ubiquitin-conjugating enzyme ubcM2, RAP1A (RAP1A, member of RAS oncogene family), UBE2E3 (ubiquitin-conjugating enzyme E2 E3), and ACTA1 (actin, alpha 1). Among them, downregulation of ubcM2, an inhibitor of Nrf2, can lead to the higher activity of this pathway. Since different components of cytoskeleton (ACTG2 and ACTA1) behave differentially and considering the lack of significant differential expression in Nrf2 or Keap1, it can be concluded that the exposure to DEPe may induce the rearrangement of the cytoskeleton (in favor of Nrf2 translocation to the nucleus) and activation of the Nrf2 pathway indirectly via oxidative stress stimuli (Fig. 4).

These findings are well supported by our transcriptomic analysis, which shows the upregulation of PRDX, GSR, and GST (downstream genes). However, the transcriptome analysis further detailed that c-Fos and FRA1 (two inhibitors of nuclear Nrf2) are significantly downregulated and therefore allow for higher Nrf2 activity during the DEPe treatment.

### Phagosome maturation

Phagosome maturation facilitates the trafficking of internalized particles toward a series of increasingly acidified, membrane-bound subcellular structures, resulting in particle degradation. After sealing or scission from the surface membrane, the phagosome is fused with early endosomes, late endosomes, and lysosomes accompanied by significant acidifying changes in its composition, converting it to an oxidative and degradative milieu. The phagosome movement occurs based on cytoskeletal protein binding (microtubules), leading to the fusion of endosomes and lysosomes sequentially in a dynamic manner (Gotthardt et al. 2002).

During the maturation, the GTPase Rab5 drives the fusion of early endosomes via interaction with several molecules, including the early endosome antigen 1 (EEA1), VPSS34 complex, and SNARE proteins. Moreover, acidification occurs simultaneously via vATPase (a vacuolar proton pump) that translocates H + across the membrane and further acidifies the lumen (Desjardins et al. 1994).

Although no significant involvement of this pathway was found at the transcriptomic level, our proteomic analysis shows downregulation of ATP6V1A (ATPase H + transporting V1 subunit A), ATP6V1E1, ATP1B2B, ATP1B3A, ATP6V1F, DYNLRB1 (dynein light chain roadblock-type 1), TUBA1C (tubulin alpha 1c), TUBB2A, TUBB4A, and VAMP2 (vesicle-associated membrane protein 2) which suggest that the treatment with DEPe may lead to lower phagosome maturation activity or altered lysosomal pH (Fig. 4). This is consistent with our recent report that DEPe exposure results in lower neuronal autophagic flux and suggests a mechanism underlying neuronal toxicity of air pollution (Barnhill et al. 2020).

### Amyloid processing

Amyloid plaques are hallmarks of neuropathological brain lesions in AD and PD with dementia. These structures are composed of A-beta peptide, which is central to the pathophysiology of AD (Sadigh-Eteghad et al. 2015). Amyloid beta is processed by gamma-secretase complex (Presenilin1/2) and beta-secretase (BACE1) from the Type 1 transmembrane protein-Amyloid protein (APP). It is known that abnormal Ca2 + influx leads to the aberrant activation of Calpain, which plays a role in the phosphorylation of the microtubule-associated protein Tau via the CDK5 pathway. Furthermore, oxidative stress associated with the accumulation of A-beta contributes to the activation of several kinases, including MAP kinases, resulting in the phosphorylation of tau. The pathological hyperphosphorylation of tau has an essential role in the destabilization of microtubules (Sadigh-Eteghad et al. 2015). These changes are particularly relevant to the pathogenesis of AD and PD and could be a contributing factor in how air pollution increases risk.

Our expression analyses suggest a destabilizing impact of DEPe on the regulators of amyloid processing as three regulators including CAPNS1 (calpain small subunit 1), MARK1 (microtubule affinity regulating kinase 1), and PRKAR1B (protein kinase cAMP-dependent type I regulatory subunit beta) were downregulated at the protein level (Fig. 4).

### Mitochondrial dysfunction

As the main consumers of oxygen in a cell, mitochondria contain a multitude of redox carriers to transfer electrons to oxygen, which results in the formation of reactive oxygen species (ROS). These organelles contain an antioxidant defense system to detoxify ROS and avoid oxidative damage to other cellular components. DEPe exposure led to changes in the expression of key proteins that likely results in mitochondrial dysfunction. As shown in Fig. 4, there is a significant decline in the protein level of ATP synthase (ATP5F1D, ATP5mea), Cytochrome c oxidase (COX7A2), NADH dehydrogenase (NDUFA10, NDUFS8), 2-oxoglutarate dehydrogenase (OGDH), and Cytochrome b-c1 complex subunit 2 (UQCRC2) that play pivotal roles in electron transportation. This could alter ATP production and also directly result in elevated ROS production and toxicity. Mitochondrial dysfunction has been proposed to contribute to the pathogenesis of AD and PD and, as with some of the aforementioned pathways, air pollution might alter risk by disrupting the function of this critical organelle.

### Axonal guidance signaling

The formation of neuronal connections requires the extension of axons that migrate toward their synaptic targets. At the axon leading edge, the axonal growth cone contains receptors with the ability to sense attractive and repulsive guidance cues that are required for the navigation of the axon. In addition to BMP, Shh, and Wnt (that have been recently implicated in axonal guidance), there are at least four major groups of guidance cues: (i) Netrins and DCC/UNC-5 receptors; (ii) Plexin, semaphorins, and neuropilin receptors; (iii) Slits and Robo receptors; and (iv) Ephrins and Eph receptors. These proteins may either be secreted and associated with the extracellular matrix (Slits, Netrins, and some Semaphorins) or be anchored to the cell surface (Ephrins and some other Semaphorins) (Stoeckli 2018).

Treatment with DEPe impacts several aspects of axonal guidance. At the protein level, a number of important effectors and regulators were altered upon the treatment. The overrepresentation of PLXNB2 (plexin B2) and underrepresentation of several proteins including ARHGEF11 (Rho guanine nucleotide exchange factor 11), CHMP1A (charged multivesicular body protein 1A), COPS5 (COP9 signalosome subunit 5), GNAT1 (G protein subunit alpha transducin 1), GNAT2 (G protein subunit alpha transducin 2), GNB5 (G protein subunit beta 5), MYL1 (myosin light chain 1), MYL2 (myosin light chain 2), NCK1 (adaptor protein 1), PLXNA1 (plexin A1), PRKAR1B (protein kinase cAMP-dependent type I regulatory subunit beta), RAC3 (Rac family small GTPase 3), RAP1A (a member of RAS oncogene family), SHANK2 (SH3 and multiple ankyrin repeat domains 2), TUBA1C (tubulin alpha 1c), TUBB2A (tubulin beta 2A class IIa), and TUBB4A (tubulin beta 4A class Iva) suggest a significant impairment of axon guidance in the developing embryo (Supplementary Table 5). Interestingly, the axonal guidance pathway was not significantly affected at the transcriptomic level, which suggests that the involvement of this pathway may be a downstream outcome through which some of the effector/regulator proteins have been degraded. Changes in axonal guidance pathways described here might reflect neurotoxic pathways relevant to AD and PD or simply reflect interference of neural development in developing embryos. Analysis of expression profiles in DEPe-exposed adults would help resolve this question.

### Other pathways

Our proteomic and transcriptomic analyses also demonstrated the involvement of a number of pathways and metabolic routes including melatonin degradation, circadian rhythm, sertoli cell-sertoli cell junction signaling, epithelial adherens junction signaling, thyroid hormone metabolism II (via conjugation and/or degradation), RhoA signaling, inosine-5′-phosphate biosynthesis II, GP6 signaling pathway, RhoGDI signaling, extrinsic prothrombin activation pathway, and coagulation system. Many of these changes could reflect alterations due to the interruption of development. For example, both proteomic and transcriptomic analyses revealed that the phototransduction pathway has the largest changes in expression upon DEPe treatment. As shown in Supplementary Table 5, several genes, including Opsin, GRK, S-arrestin, Transducin-α, Transducin-γ, PDC, and GUCA have an attenuated level of transcription due to the exposure to DEPe. Due to the lack of evidence of induction of any element in this pathway, DEPe treatment appears to have a negative effect on the development of the visual system. This is consistent with our observation that the eyes never fully developed and are small following the treatment.

### Functional assessment

**Fig. 5:**
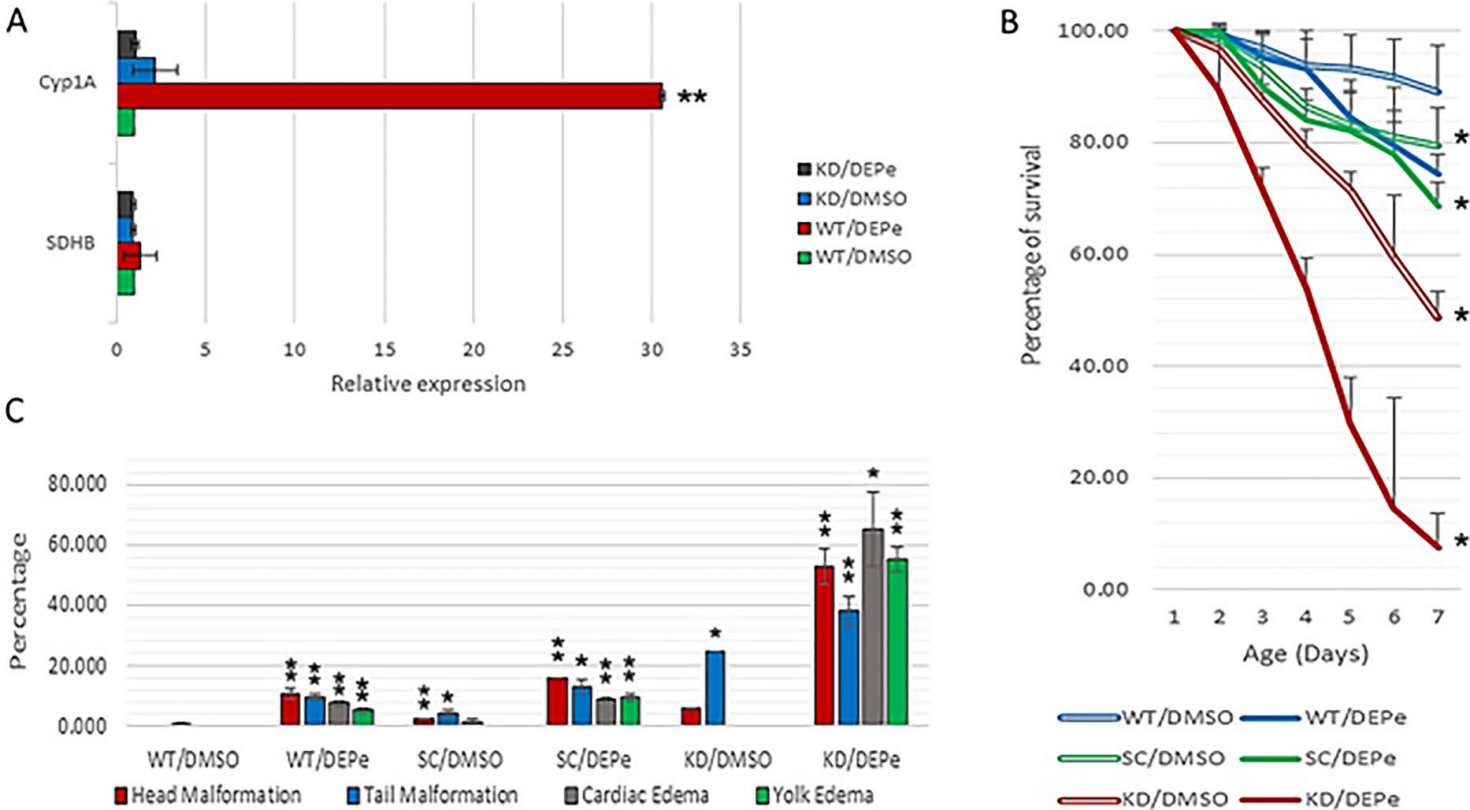
Cyp1A knockdown model. **A** Lack of induced expression of Cyp1A confirmed the KD efficiency; the expression levels were normalized to Elf-alpha as an internal control. Additionally, from the RNA-seq results table, the succinate dehydrogenase complex subunits B (SDHB) with stable expression between groups was selected as a secondary internal control. **B** The KD embryos had decreased survival in both treated and control conditions, but there was a significantly increased vulnerability to DEPe in KD embryos compared to controls. **C** Increased rate of morphological defects in KD embryos after exposure to DEPe demonstrates increased vulnerability in the absence of Cyp1A. The asterisks indicate statistical significance (∗ ∗  = p < 0.01, ∗  = p < 0.05)

In this report, the significant upregulation of cytochrome P450 (Cyp1A) was the most prominent effect of DEPe exposure. Cyp1A metabolizes many endogenous and exogenous compounds and increased expression in the brain may be protective against, or contribute to, toxicity. To better understand the role of Cyp1A in DEPe toxicity, we utilized CRISPR/Cas9 to generate a Cyp1A knockdown model. Since the gene-editing procedure in zebrafish is likely to be a partial process resulting in mosaic embryos, we use the term “knock down” (KD) instead of “knock out.” Using a sequence-specific guide-RNA, we converted the first CCA PAM site to a TGA (stop) codon in the first exon of Cyp1A. A non-target guide-RNA was alternatively used as scramble control (SC). Total RNA was then isolated from the heads of up to 20 KD and SC embryos (5 dpf), and the knockdown efficiency was validated by qPCR analysis. Similar to what was observed in the high throughput RNA-Seq study, this experiment demonstrated a sharp induction (30.59-fold from the baseline) of Cyp1A upon DEPe treatment in the SC embryos, while the KD embryos lacked such an increase in expression (Fig. 5a). The survival of six groups of embryos during a 7-day time course (n ≥ 40) is shown in Fig. 5b. Briefly, at 7 dpf, the average survival was 89.1%, 74.4%, 79.4%, 68.5%, 48.8%, and 7.6% for the WT/DMSO, WT/DEPe, SC/DMSO, SC/DEPe, KD/DMSO, and KD/DEPe, respectively. Thus, KD of Cyp1A resulting in reduced survival but was dramatically reduced in the DEPe-treated fish compared to controls. Furthermore, KD also resulted in a significantly higher rate of morphological malformations (Fig. 5c). Thus, the induction of Cyp1A expression by DEPe appears to be a protective compensatory mechanism. A significant limitation of our findings is that we measured Cyp1A induction of expression in the zebrafish brains but KD experiments reduced expression in the entire embryo.

### Importance and implications for neurodegenerative diseases

Air pollution is known to negatively impact the quality of life and play important roles in the development of human and animal diseases. Using high throughput expression analyses, we showed that DEPe treatment altered several biological processes and signaling pathways in the heads of zebrafish embryos heads. Alterations in phagosome formation, amyloid processing, and mitochondrial function are particularly relevant to neurodegeneration. Surprisingly, our results did not indicate any clear involvement of inflammation, which has been hypothesized to play an important role in air pollution toxicity. This is likely due to the fact that we measured expression only in the heads of 5-day old zebrafish and not the entire body.

The intent of these studies was to determine how the brain is impacted by exposure to DEPe. The brain does contain microglia, which likely are activated by DEPe, but they compose such a small fraction of the cells in the brain that any changes may be undetectable. In addition, microglia are not present and functioning in the brain of developing larvae until 3–5 dpf. Because of this, early exposure may not be the most relevant for studying inflammatory responses. Induction of xenobiotic metabolism is also likely relevant as a compensatory or protective response to air pollution. Our Cyp1A KD model validated the protective role of this pathway and represents an excellent example of how expression studies can provide important clues to determine mechanisms of toxicants such as air pollution. It is not known if polymorphisms in the Cyp1A gene alters the risk of AD or PD in humans exposed to air pollution, but these results suggest a potential gene-environment interaction and deserve further investigation.

One important question to address is the relevance of DEPe exposures to air pollution. DEPe is the most common mixture used in air pollution research for cell culture and other models that are not amenable to inhalation chambers. The dichloromethane extracts of DEP contain hydrophobic moieties such as PAHs that are most likely to enter the bloodstream and bioaccumulate in the brain. A recent study determined the concentration of several PAHs in human autopsy brains and found that they contained concentrations of PAHs very similar to those used in the studies presented here (Pastor-Belda et al. 2019). Thus, the exposures to zebrafish brains are very relevant, as they contain PAHs and other moieties that have been observed to bioaccumulate in the human brain.

## Supplementary Information

Below is the link to the electronic supplementary material.

Supplementary file1 (XLSX 10 KB)

Supplementary file2 (XLSX 1329 KB)

Supplementary file3 (XLSX 152 KB)

Supplementary file4 (XLSX 4167 KB)

Supplementary file5 (XLSX 2356 KB)

## Data Availability

All data are available in our supplemental files.
